# Non-Targeted Metabolomic Analysis of Chicken Kidneys in Response to Coronavirus IBV Infection Under Stress Induced by Dexamethasone

**DOI:** 10.3389/fcimb.2022.945865

**Published:** 2022-07-15

**Authors:** Jun Dai, Huan Wang, Ying Liao, Lei Tan, Yingjie Sun, Cuiping Song, Weiwei Liu, Chan Ding, Tingrong Luo, Xusheng Qiu

**Affiliations:** ^1^ Laboratory of Veterinary Microbiology and Animal Infectious Diseases, College of Animal Sciences and Veterinary Medicine, Guangxi University, Nanning, China; ^2^ Shanghai Veterinary Research Institute, Chinese Academy of Agricultural Sciences, Shanghai, China; ^3^ Jiangsu Co-innovation Center for Prevention and Control of Important Animal Infectious Diseases and Zoonoses, Yangzhou University, Yangzhou, China; ^4^ State Key Laboratory for Conservation and Utilization of Subtropical Agro-Bioresources, Guangxi University, Nanning, China

**Keywords:** dexamethasone, stress, non-targeted metabolomic, coronavirus, IBV

## Abstract

Stress in poultry can lead to changes in body metabolism and immunity, which can increase susceptibility to infectious diseases. However, knowledge regarding chicken responses to viral infection under stress is limited. Dexamethasone (Dex) is a synthetic glucocorticoid similar to that secreted by animals under stress conditions, and has been widely used to induce stress in chickens. Herein, we established a stress model in 7-day-old chickens injected with Dex to elucidate the effects of stress on IBV replication in the kidneys. The metabolic changes, immune status and growth of the chickens under stress conditions were comprehensively evaluated. Furthermore, the metabolic profile, weight gain, viral load, serum cholesterol levels, cytokines and peripheral blood lymphocyte ratio were compared in chickens treated with Dex and infected with IBV. An LC-MS/MS-based metabolomics method was used to examine differentially enriched metabolites in the kidneys. A total of 113 metabolites whose abundance was altered after Dex treatment were identified, most of which were lipids and lipid-like molecules. The principal metabolic alterations in chicken kidneys caused by IBV infection included fatty acid, valine, leucine and isoleucine metabolism. Dex treatment before and after IBV infection mainly affected the host’s tryptophan, phenylalanine, amino sugar and nucleotide sugar metabolism. In addition, Dex led to up-regulation of serum cholesterol levels and renal viral load in chickens, and to the inhibition of weight gain, peripheral blood lymphocytes and IL-6 production. We also confirmed that the exogenous cholesterol in DF-1 cells promoted the replication of IBV. However, whether the increase in viral load in kidney tissue is associated with the up-regulation of cholesterol levels induced by Dex must be demonstrated in future experiments. In conclusion, chick growth and immune function were significantly inhibited by Dex. Host cholesterol metabolism and the response to IBV infection are regulated by Dex. This study provides valuable insights into the molecular regulatory mechanisms in poultry stress, and should support further research on the intrinsic link between cholesterol metabolism and IBV replication under stress conditions.

## Introduction

Animal stress is a complex, multidimensional and universal phenomenon that is difficult to evaluate and has important biological significance (Buwalda et al., 2012). Poultry stress is a global problem that causes large economic losses and threatens poultry health and welfare (Yang et al., 2014; Zaboli et al., 2019). Multiple stressors are present during the growth of chickens, including immune stress, oxidative stress, transport stress and cold or heat stress (Sun et al., 2018; Guo et al., 2020; Goel et al., 2021). Compared with mammals, chickens have a higher body temperature and are extremely sensitive to temperature changes during the breeding process (Lara and Rostagno, 2013; Collier and Gebremedhin, 2015), particularly in cold regions (Blahová et al., 2007). Cold or heat stress is the most common environmental stressor in chickens, and may cause pathophysiological stress (Castellani and Young, 2016; Lin et al., 2006; Wang et al., 2018; Hu and Cheng, 2021). For example, cold or heat stress not only affects tissue inflammatory factor levels (Zhao et al., 2013; Zhang et al., 2014) but also causes severe damage to the liver, heart and intestinal tissues in chickens (Zhang et al., 2011; Wei et al., 2018). Moreover, stress has been found to significantly increase susceptibility to necrotic enteritis and enhance bacterial translocation in a subclinical experimental model (Tsiouris et al., 2015; Borsoi et al., 2015; Zhou et al., 2021). Evidence has indicated that cold stress decreases immune function in animals, and temperature downshifts can make animals susceptible to viral infection. For example, outbreaks of H1N1 (Steel et al., 2010), RSV (Zhang et al., 2013; Donaldson, 2006; Noyola and Mandeville, 2008; Tang, 2009), PEDV (Kong et al., 2020), NDV (Pfitzer et al., 2000) and RV (Foxman et al., 2015) usually occur in cold winter seasons. Previous studies on stress in poultry have focused on changes in cerebellar development (Austdal et al., 2016), energy intake (Benson et al., 1993; Garriga et al., 2006; Raghebian et al., 2016; Hu et al., 2021), growth (Nawab et al., 2018; Chen et al., 2021; Kumar et al., 2021), inflammatory responses (Wein et al., 2017; Lan et al., 2020; Liu et al., 2021), oxidative responses (Gao et al., 2010; Jiao et al., 2018; Pan et al., 2019) and immunosuppression (Guo et al., 2020; Guo et al., 2021; Su et al., 2021). However, the detailed molecular mechanisms linking stress and viral replication remain unclear.

Studies have indicated that the hypothalamic-pituitary-adrenal (HPA) axis is an important system for integrating and regulating stress responses *in vivo* and *in vitro* (McEwen, 2000; Iyasere et al., 2017; Beckford et al., 2020). In general, various stressors can cause changes in certain HPA axes that are required for stress adaptation (Scheff et al., 2012). Activation of the HPA axis and sympathetic nervous system when chickens are exposed to stress induces the rapid secretion of glucocorticoids (GCs) from the adrenal glands (Chrousos and Gold, 1992; Aguilera, 1994; Lin et al., 2006; Chrousos, 2009; Borsoi et al., 2015; Scanes, 2016). GCs are a class of steroid hormones that respond rapidly to environmental and physiological stimuli (Carsia et al., 1988; Revollo and Cidlowski, 2009; Alba et al., 2019; Häffelin et al., 2020). Excessive GCs are a sign of stress (Chrousos and Gold, 1992; Webster Marketon and Glaser, 2008; Quinteiro-Filho et al., 2017). Through the release of GCs, the HPA axis mobilizes energy reserves, thus ensuring that the organism has sufficient resources to respond to actual bodily harm or to prepare for anticipated harm and alleviate the adverse effects (Herman et al., 2016; Zheng et al., 2021). Additionally, GCs, the final product of the HPA axis, are cholesterol-derived molecules that exert pleiotropic and nongenomic effects through the glucocorticoid receptor (GR) (Chrousos and Kino, 2007). GCs also plays an important role in maintaining resting and stress-associated homeostasis and affects the body’s physiological adaptive response to stressors (Nicolaides et al., 2015). The secretion of GC is a typical stress endocrine response (Sapolsky et al., 2000; Flinchum and Gwen, 2015) that is particularly important in poultry stress biology (Scanes, 2016). In poultry, two of the most common physiological parameters of stress are circulating concentrations of the adreno-cortical hormone corticosterone (CORT) and the heterophil:lymphocyte ratio (H:L) (Kalliecharan and Hall, 1974; Scanes, 2016). CORT is the main poultry glucocorticoid that regulates energy reserves to meet metabolic demands. Poultry stress causes elevated plasma CORT, stimulates glucocorticoid receptors, and may promote glycemia, lipolysis and proteolysis (Deviche et al., 2017). Therefore, proper control of the stress response is critical for the body, because inappropriate or prolonged activation of the HPA axis is energy-draining and has been implicated in many physiological disease states (Myers et al., 2012). Dex is a synthetic glucocorticoid similar to that secreted by animals under stress conditions (Lin et al., 2004; Gao et al., 2008), which has been used in many studies as a glucocorticoid mimic (Chang et al., 2015). In particular, Dex is widely used in the establishment of chicken stress models (Eid et al., 2006; Gao et al., 2010; El-Senousey et al., 2018; Pan et al., 2019; Zhou et al., 2019; Zhai et al., 2020; Osho and Adeola, 2020; Su et al., 2021). Therefore, Dex is a satisfactory stress-inducing drug that provides an effective way to study the relationship between stress and viral replication.

Herein, we established a chicken stress model by using Dex and found that Dex-induced chicken stress increased the IBV viral load in the kidneys. Untargeted metabolomics was used to detect differentially enriched metabolites in chicken kidney tissue to evaluate the correlations between IBV replication and metabolic changes induced by Dex. Serum cholesterol levels significantly increased in chickens under stress. Many metabolic pathways and metabolites may be associated with the replication and pathogenicity of IBV. This finding provides insight into the intrinsic link between host metabolism and IBV replication under stress conditions.

## Materials and Methods

### Cell Culture and Virus

DF-1 (ATCC, CRL-12203), Vero and H1299 cell lines were obtained from the American Type Culture Collection (ATCC) (Wang et al., 2019; Wang et al., 2021). The IBV QX and Beaudette strains (ATCC VR-22) were kept at the Shanghai Veterinary Research Institute, Chinese Academy of Agricultural Sciences, Shanghai (Wang et al., 2021; Gao et al., 2021). The IBV Beaudette strain used in this study was adapted to DF-1, H1299 and Vero cells, and was a gift from Prof. Dingxiang Liu**’**s laboratory (South China Agricultural University) (Wang et al., 2019; Gao et al., 2021). The culture of DF-1, Vero and H1299 cell lines was performed according to previously described methods in our laboratory (Wang et al., 2019; Liu et al., 2019; Gong et al., 2021), In brief, DF-1 and Vero cells were grown in Dulbecco**’**s modified Eagle**’**s medium with 10% fetal calf serum. H1299 cells were maintained in Roswell Park Memorial Institute 1640 medium supplemented with 10% (v/v) fetal calf serum. The above cells were cultured at 37 °C under 5% CO_2_.

### Antibodies, Reagents and Chickens

Anti-IBV N antibody was obtained by immunization of a rabbit with IBV N antigen in our laboratory as previously described (Wang et al., 2021). CD4-FITC, CD8-PE and CD3-SPRD monoclonal antibodies were purchased from Southern Biotech (USA). The 25-hydroxycholesterol (HY-113134) and cholesterol (HY-N0322) were purchased from MedChemExpress. Dexamethasone sodium phosphate injection was purchased from Jiangxi Bolai Pharmacy Co., Ltd. Plasma total cholesterol (TC), high-density lipoprotein cholesterol (HDL-C) and low-density lipoprotein cholesterol (LDL-C) concentrations were assessed with an automatic biochemical analyzer (Rayto, chemray240). A lymphocyte separation kit (P8740) was purchased from Beijing Soleibao Technology Co., Ltd. ELISA kits for IFN-γ (ZC-51624), IL-1β (ZC-51658), TNF-α (ZC-51975), IL-6 (ZC-51663) and IFN-β (ZC-51619) were purchased from Shanghai Zhuo Cai Technology Co., Ltd. All specific pathogen free (SPF) embryonated eggs were purchased from Beijing Boehringer Ingelheim Vital Biotechnology Co., Ltd. (Beijing, China) and incubated as previously described (van de Ven et al., 2011; Maatjens et al., 2014; Mesquita et al., 2021), In summary, SPF embryonated eggs were incubated for 17** d** in a multi-stage incubator (science incubator TM, Shandong, China) at a constant machine temperature of 37.6°C and relative humidity of 53%. Eggs were placed with the air cell up and turned hourly at an angle of 90°. From E17 to E21.5, the eggs were not turned, the air temperature remained constant at 35.0°C, and the air speed was lower than 0.2** m/s**, which is considered still air.

### Western Blot Analysis

DF-1 cells, Vero cells and H1299 cells were harvested at the indicated infection time points and lysed with 2× SDS loading buffer in the presence of 100 mM dithiothreitol, then denatured at 100°C for 10 min. The obtained protein samples were separated with SDS-PAGE to detect intracellular IBV protein levels according to a previously described method (Wang et al., 2021).

### Animal Experiments

A total of 260 healthy White Leghorns of similar weight were randomly and averagely divided into five groups: mock (N=52), Dex (N=52), IBV (N=52), IBV-P-Dex (pretreatment with Dex before infection, N=52) and IBV-A-Dex (treatment with Dex after infection, N=52). The feeding and care of chickens was performed in accordance with the Institutional Animal Care and Use Committee guidelines. The approval number was SHVRI-chicken-2022021802. According to previous studies (Zhou et al., 2019; Guo et al., 2020; Su et al., 2021), a chicken stress model was established through subcutaneous injection of Dex (2.0 mg/kg, once/day), and PBS was used as a negative control. Seven-day-old SPF chickens were challenged *via* an eye dropper with 200 μl of 10^5^ EID50 of IBV-QX or PBS as the negative control. Chickens were observed daily, and their clinical symptoms and mortality were recorded. At 7 dpi (14 days old), the chickens’ responses to IBV infection under stress induced by Dex were comprehensively assessed through measurement of viral load, histopathology, metabolomics, serum cholesterol concentration, peripheral blood lymphocyte ratio, survival rate and growth performance (**Supplementary Materials Figure S1** shows the experimental design).

### Histopathology

The trachea, heart, lung, liver, kidney and spleen tissues were isolated and soaked in 10% neutral formalin at room temperature for more than 48 h, then subjected to routine processing. H&E staining was performed, and the slices of organs for each chicken were observed under an optical microscope.

### Sample Preparation and Extraction

Samples were thawed on ice, and 50 mg tissue fragments were taken from each sample and mixed with 500 μl ice-cold methanol/water (70%, v/v). After homogenization at 30 Hz for 2 min, the mixture was vibrated for 5 min and incubated on ice for 15 min. After centrifugation at 12,000 rpm at 4°C for 10 min, 400 μl supernatant was transferred into another centrifuge tube; subsequently, 500 μl of ethyl acetate/methanol (V, 1:3) was added into the original centrifuge tube. The mixture was oscillated for 5 min, incubated on ice for 15 min and centrifuged at 12,000 rpm at 4°C for 10 min, and 400 μl of supernatant was collected. The two supernatants were combined and concentrated. Then 100 µl of 70% methanol water was added to the dried product, and ultrasonic treatment was performed for 3 min. Finally, samples were centrifuged at 12,000 rpm at 4°C for 3 min, and 60 µl of supernatant was aspirated for LC-MS/MS analysis.

### Metabolomics Analysis

Untargeted metabolomics profiling was performed using a UPLC-Q-TOF/MS (AB SCIEX, MA, USA). The chromatographic seperation system was equipped with a Waters ACQUITY UPLC HSS T3 column (1.8 um, 2.1 mm *100 mm, Waters Co.). UPLC conditions were as follow: column temperature, 40°C; flow rate, 0.40 mL/min; injection valume, 1ul. The mobile phase consisted 0.1% formic acid inwater (phase A), and 0.1% formic acid in acetonitrile (phase B). The linear gradient programm was as follows: 95% to 10% phase A over 11 min, and holding 10% for 1 min, and 10% to 95% in 0.1 min, and holding 95% phase A for 1.9 min. The mass spectrometer was operated in positive/negative polarity mode with the following settings: Nitrogen was used as the drying gas, nebulizer gas and sheath gas, and the flow was maintained at 8 L/min, while sheath flow was at 11 L/min. The drying gas and sheath gas temperature was maintained at 325°C. The ESI+ and ESI- voltages were set at 2500 V and 1500 V respectively. Mass range was set at m/z 50-1700 and the resolution was 30,000 (FWHM). The mass spectrometer was calibrated daily in the mass range m/z 100-1700 before starting the sample analysis by using Agilent tune mix (Part no G1969-85000). The mass accuracy values were good in full scan range (mass error < 5 ppm).

### Quantitative Real-Time PCR Analysis

Total RNA from kidneys was used for quantitative real-time PCR (qPCR) analysis to confirm the propagation of the IBV in chicks, and the mock group was used as a negative control. The primer pairs used in the PCR assay for the IBV-N gene were IBV Fw-5′-CAG AAG AAG GGC TCT CGC ATT AC-3′ and IBV Re-5′-AGG TTG AGC ATT GCC GTA ACA C-3′. Positive and negative strands of IBV genomic RNA were detected according to our previous report (Liao et al., 2011). The qPCR specific primers for ch-DHCR24 and ch-CH25H were designed with reference to previous literature (Liu et al., 2019). RNA was extracted with an RNeasy Mini Kit (QIAGEN, ID: 74104, Germany) according to the manufacturer’s protocols. Validation of RNA-seq data by qPCR was performed as previously described (Liu et al., 2018).

### Statistical Analysis

The original data file obtained by LC-MS analysis was first converted into mzML format in Proteo Wizard software. Peak extraction, alignment and retention time correction were performed with the XCMS program. The “Support Vector Regression (SVR)” method was used to correct the peak area (Vapnik et al., 2008; Al-Zoubi et al., 2011; Sánchez-Illana et al., 2018). The peaks were filtered with a deletion rate > 50% in each group of samples. Metabolic identification information was then obtained by searching the laboratory’s self-built database (Wuhan Metware Biotechnology Co., Ltd.) and integrating the public database and metDNA. Finally, statistical analysis was performed in the *R* program (**Supplementary Materials Table S1**). Statistical analysis included univariate analysis and multivariate analysis, and univariate statistical analysis included Student’s t-test and variance multiple analysis. Multivariate statistical analysis included principal component analysis (PCA), partial least squares discriminant analysis (PLS-DA) and orthogonal partial least squares discriminant analysis (OPLS-DA). The identification of differentially enriched metabolites and metabolic pathways was as previously described (Liu et al., 2019).

## Results

### Dex Induces Weight Loss in Chickens and Promotes IBV Replication *In Vivo*

**Figure 1:**
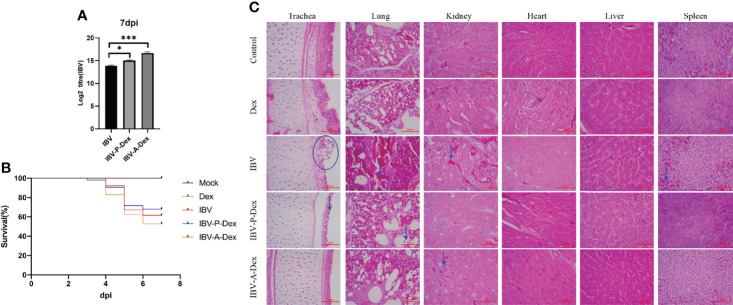
Dex promotes IBV replication *in vivo*. **(A)** Assessment of IBV viral load in chicken kidney tissues by qPCR. **(B)**. Survival curve showing the survival percentage in each group within the 7dpi observation period. **(C)**. Histology staining of the trachea, lungs, kidneys, heart, liver and spleen in chickens from the five groups at 7 dpi (HE, 400×). The blue arrows and circles indicate areas of tissue lesions.

To evaluate the effects of Dex treatment and IBV infection on chicken growth, we weighed chickens at 1, 7 and 14 days of age. As shown in the **Supplementary Materials Table S2**, the body weights of the Dex and IBV groups were significantly lower than that of the control at 14 days of age. Thus, both Dex treatment and IBV infection inhibited chicken weight gain. To confirm whether Dex affected IBV replication *in vivo*, we assessed the effect of Dex on IBV replication, on the basis of chicken mortality and IBV viral load in kidney tissue (**Figures 1A, B**). The IBV viral loads in the IBV-P-Dex group and the IBV-A-Dex group were higher than that in the IBV group. Dex promoted the replication of IBV in chicken kidneys but did not cause a significant difference in mortality. In addition, we used pathological slides to assess whether Dex affected IBV pathogenicity. The trachea, lung, kidney, heart, liver and spleen tissues were subjected to histopathological observation with hematoxylin and eosin staining. All chickens infected with IBV-QX strains showed different degrees of pathological changes in the trachea, lung, kidneys and spleen, but not the heart and liver tissues (**Figure 1C**). No clear pathological changes were found in the control and Dex groups. Histological examination revealed substantial damage to the tracheal mucosal cortex: the mucosal cells were necrotic and shedding, and a small amount of inflammatory cells had infiltrated. Many mass hemorrhages were observed in the bronchial cavities and pulmonary chambers of the lungs, filling the cavities. Clear renal interstitial hyperemia was observed, and the renal tubular epithelial cells were swollen and degenerated. The splenic sinus was dilated and congested or bleeding in the red pulp area of the spleen, and a small amount of heterophilic granulocyte infiltration was observed. These results indicated that the IBV-QX strain successfully infected chickens and showed strong tropism for kidney and tracheal tissue, in agreement with findings from previous studies (Bouwman et al., 2019; Laconi et al., 2020).

### Dex Induces Immunosuppression

**Figure 2:**
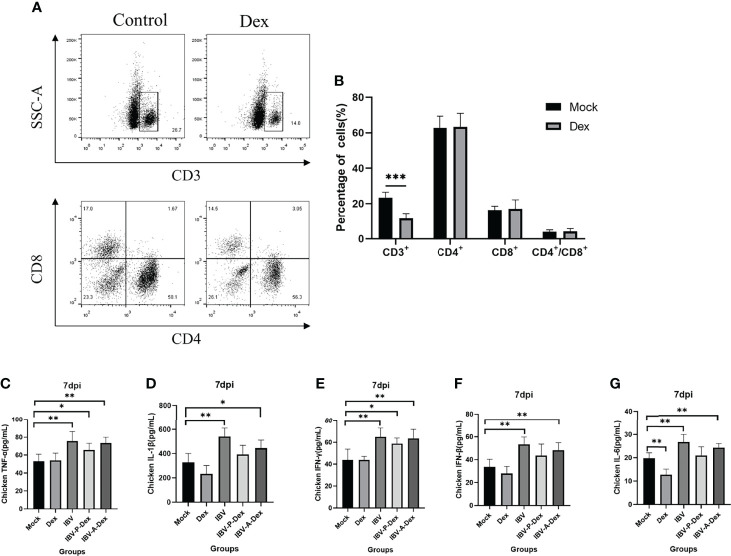
Dex induces immunosuppression. **(A)**. Lymphocytes were isolated with a lymphocyte separation kit (P8740). CD4-FITC, CD8-PE and CD3-SPRD monoclonal antibodies were added and reacted at room temperature in the dark for 30 minutes. Resuspended cells were detected with a flow cytometer (Bio-Rad, ZE5, USA). **(B)**. The percentage of peripheral blood lymphocytes, analyzed with Cell Quest software. Plasma TNF-α **(C)**, IL-1β **(D)**, IFN-γ **(E)**, IFN-β **(F)** and IL-6 **(G)** levels were assessed with an ELISA kit and standard curve (**Supplementary Materials Figure S2**).

Chicken stress can lead to immunosuppression and metabolic alterations, which can consequently increase susceptibility to infectious diseases (Wang et al., 2020; Kikusato et al., 2021). Therefore, we detected the percentage of CD3^+^ T cell subsets and cytokines in the peripheral blood to determine the immune status of the chickens. As shown in **Figures 2A, B**, the percentage of CD3+ T cell subsets in the Dex group was significantly lower than that in the control. ELISA indicated that the levels of the serum cytokines TNF-α, IL-1β, IFN-γ, IFN-β and IL-6 in chickens were significantly higher at 7 dpi after infection with IBV than in the control (**Figures 2C–G**). In addition, with Dex intervention, the cytokine levels in the IBV-P-Dex and IBV-A-Dex groups were lower than those in the IBV group (**Figures 2C–G**). These results revealed that Dex causes immunosuppression in chickens; however, whether immunosuppression is the main reason for the promotion of IBV replication remains unclear.

### Multivariate Analysis of Chicken Kidney Metabolites

**Figure 3:**
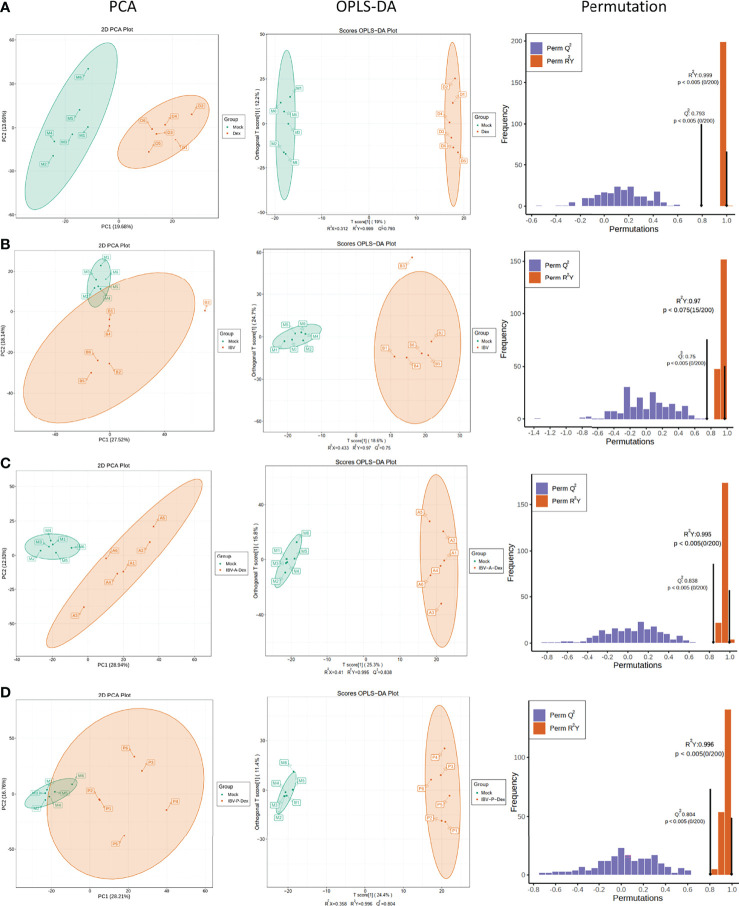
Multivariate analysis of chicken kidney metabolites. Metabolic profile of the mock and Dex group **(A)**, IBV group **(B)**, IBV-A-Dex group **(C)** and IBV-P-Dex group **(D)**, visualized by PCA, OPLS-DA analysis and a permutation test. Ellipses represent 95% confidence intervals. **(A–D)** were derived from POS and **Supplementary Materials Figure S3** was derived from NEG.

To investigate the metabolic pathway changes associated with Dex treatment and IBV infection, we used an LC-MS/MS-based metabolomics method to examine differentially enriched metabolites in the chicken kidneys. Metabolite identification information was obtained by searching laboratory-built databases, and integrating public libraries and met-DNA methods. To comprehensively collect reliable information on the mock and other groups (mock vs Dex, mock vs IBV, mock vs IBV-A-Dex, and mock vs IBV-P-Dex), we used the principle component analysis (PCA) to compare the metabolite composition (**Figures 3A–D**). OPLS-DA models were used to determine whether Dex affected metabolic patterns, and a permutation test was further applied to validate the accuracy and predictive ability of the OPLS-DA model (**Figures 3A–D**). The results of PCA and OPLS-DA analysis showed a clear separation between the content of the control and other groups, thus indicating that significant changes in the concentrations of metabolites in the kidneys were induced by Dex and IBV infection, which could be used for subsequent studies.

### Lipid Synthesis Is Regulated by Dex and IBV Infection in Chicken Kidneys

**Figure 4:**
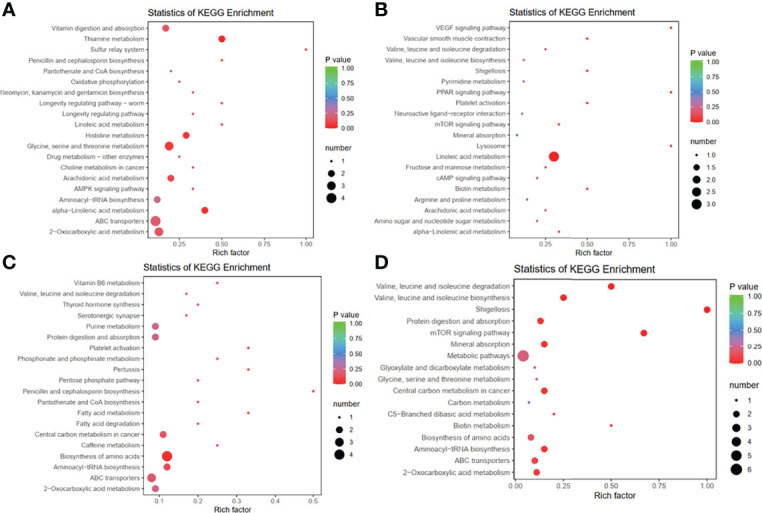
Dex treatment and IBV infection alters metabolic profiles in the kidneys of chickens. (A, B). Bubble plots of the metabolic pathway analysis in chickens after Dex treatment (Mock vs Dex). **(A)** was derived from POS, and **(B)** was derived from NEG. **(C, D)**. Bubble plots of the metabolic pathway analysis for chickens after IBV infection (Mock vs IBV). **(C)** was derived from POS, and **(D)** was derived from NEG. Each bubble represents a metabolic pathway. The x-axis represents the ratio of the number of differentially enriched metabolites in corresponding pathways to the total number of metabolites detected in the pathway. The y-axis indicates different metabolic pathways in the enrichment analysis, and the color of the point is the p value.

The differentially enriched metabolites between the control and Dex groups were key to explaining the occurrence of stress in chickens induced by Dex. The combination of fold change (fold change ≥ 2 and fold change ≤ 0.5), P value (<0.05) and VIP (≥1) in the OPLS-DA model was used to screen for differentially enriched metabolites, and a total of 147 differentially enriched metabolites were obtained in the Dex group. The lists of differentially enriched metabolites are shown in **Supplementary Materials Table S3** (POS) and **Table S4** (NEG), and were visualized *via* a volcano plot (**Supplementary Materials Figures S4A, B**) and hierarchical clustering (**Supplementary Materials Figures S5A, B**). A total of 113 renal metabolites (74 up-regulated, 39 down-regulated) derived from POS significantly changed after Dex treatment, most of which were involved in lipid metabolism pathways. Approximately 39.19% (29/74) of the 74 up-regulated metabolites were lipids and lipid-like molecules, according to the class I identification. According to the class II identification, fatty acyls (13/74), glycerophospholipids (6/74), carboxylic acids and derivatives (6/74), organonitrogen compounds (6/74), and steroids and steroid derivatives (5/76) were the top five metabolites. Of the 39 down-regulated metabolites, 43.59% (17/39) were lipids and lipid-like molecules, of which glycerophospholipids (7/39), glycerolipids (3/39), prenol lipids (3/39), and steroids and steroid derivatives (3/39) were the top three metabolites, according to class II (**Supplementary Materials Table S3**). In summary, Dex may be involved in the regulation of host lipid metabolism, affecting the biosynthesis of fatty acyls, glycerolipids, prenol lipids, steroids and glycerophospholipids. In addition, the KEGG enrichment analysis results revealed that the differentially enriched metabolites participated in nine target pathways including vitamin digestion and absorption; linoleic acid and alpha-linolenic acid metabolism; thiamine, histidine and arachidonic acid metabolism; and glycine, serine and threonine metabolism (**Figures 4A, B**). These pathways are associated with amino acid metabolism and fatty acid metabolism, which are involved in protein and lipid synthesis, respectively.

We used PCA analysis, OPLS-DA model analysis, permutation tests (**Figure 3B** and **Figure S3B**) and KEGG pathway enrichment analysis (**Figures 4C, D**) to evaluate the changes in kidney metabolites in chicks after IBV infection. The lists of differentially enriched metabolites are shown in **Supplementary Materials Table S5** (POS) and S6 (NEG), and are visualized *via* a volcano plot (**Supplementary Materials Figures S4C, D**) and hierarchical clustering (**Supplementary Materials Figures S5C, D**). The results showed that IBV infection caused a decrease in the levels of PE-NMe (14:0/20:1(11Z)), eicosapentaenoyl PAF C-16, PA (20:0/a-15:0), PA (20:0/a-17:0), PC (18:1(11Z)/18:2(9Z,12Z)) and PA (21:0/14:0), which are involved in the biosynthesis of biofilms and bile. However, approximately 48% (24/50) of the 50 up-regulated metabolites were lipids and lipid-like molecules, according to the class I identification. An increase was observed in the levels of many nucleotides and derivatives, glycerophospholipids, and steroids and steroid derivatives, which are associated with cholesterol metabolism and nucleotide synthesis. The changes in these metabolites caused by IBV infection were found to contribute to viral replication.

### IBV-Induced Metabolic Responses of Chicken Kidneys Are Affected by Dex

**Figure 5:**
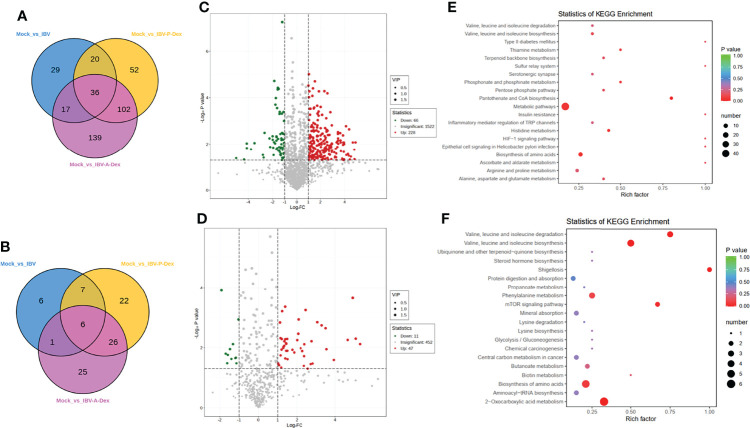
IBV-induced metabolic responses of chick kidneys are affected by Dex. Metabolite changes were further examined with a Venn diagram (A, B). **(A)** was derived from POS, and **(B)** was derived from NEG. **(C, D)** are the volcano plots for the mock and IBV-A-Dex group. Each point in the volcanic map represents a metabolite. Red: upregulation; blue: downregulation; gray: not significant. **(C)** was derived from POS, and **(D)** was derived from NEG. **(E, F)** are the KEGG pathway enrichment analyses based on differentially enriched metabolites in IBV-A-Dex group relative to the mock. **(E)** was derived from POS, and **(F)** was derived from NEG. Each bubble represents a metabolic pathway. The x-axis represents the ratio of the number of differentially enriched metabolites in corresponding pathways to the total number of metabolites detected in the pathway. The y-axis indicates different metabolic pathways in the enrichment analysis, and the color of the point is the p value.

To confirm whether the IBV-induced metabolic responses of chicken kidneys were affected by Dex, we compared the metabolic profiles of the IBV group, IBV-P-Dex group and IBV-A-Dex group with the mock group. PCA and OPLS-DA analysis showed a clear separation between the mock and IBV-A-Dex or IBV-P-Dex groups (**Figures 3C, D**, **Supplementary Materials Figures S3C, D**), thus indicating that Dex induced significant changes in the concentrations of metabolites in the kidney responses to IBV infection. The global metabolite changes in terms of their similarity and uniqueness among the three groups were further examined through a Venn diagram (**Figures 5A, B**), in which 36 metabolites were common to the three groups (**Figure 5A**). The observed number of metabolites associated with IBV infection in the IBV-A-Dex group was approximately three-fold greater than that in the IBV-P-Dex group, and 24 metabolites were common to both groups (**Supplementary Materials Figure S6A**). These results indicated metabolic differences between Dex treatment before versus after IBV infection. The lists of differentially enriched metabolites are shown in **Supplementary Materials Tables S7**–**S10**, and the volcano plots are shown in **Figures 5C, D** and **Supplementary Materials Figures S6C, D**. These significantly differentially enriched metabolites included fatty acyls, amino acid derivatives, hormones and hormone related compounds, carboxylic acids and derivatives, small peptides, and steroids and steroid derivatives, according to the class II identification. In addition, the KEGG enrichment analysis results are shown in **Figures 5E, F** and **Supplementary Materials Figures S6E, F**. The analysis revealed that the differentially enriched metabolites were involved in multiple metabolic pathways, including phenylalanine metabolism, 2-Oxocarboxylic acid metabolism, biosynthesis of amino acids, and synthesis and degradation of valine, leucine and isoleucine. These alterations in host metabolism are likely to be the underlying factors responsible for differences in the level of IBV replication in the kidneys.

### Correlation Between Cholesterol Metabolism and IBV Replication Under Stress

**Figure 6:**
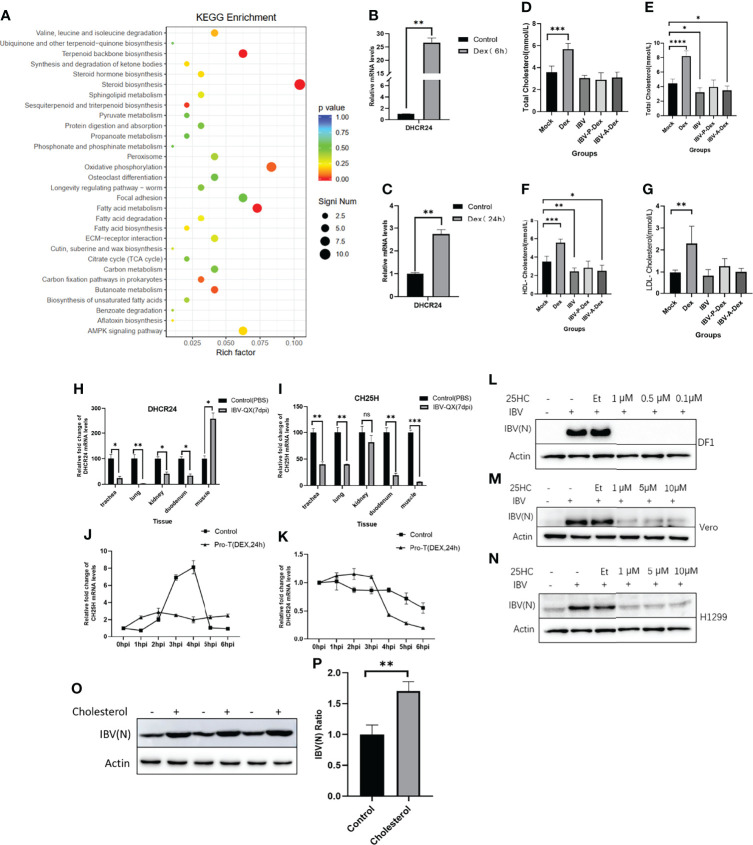
Host cholesterol metabolism is affected by Dex treatment and IBV infection **(A)** KEGG analyses based on differentially expressed genes in DF-1 cells pretreated with Dex (10 μg/mL) for 24 h relative to controls. Circles indicate numbers of genes, and colors depict the enrichment. **(B, C)**. qPCR detection of mRNA levels of DHCR24 in DF-1 cells treated with Dex (10 μg/mL) for 6 or 24 h, and the Dex-untreated group was used as a control. The gene expression was quantified relative to actin expression with the 2^-△△^CT method according to our previous research (Liu et al., 2018). **(D)**. An automatic biochemical analyzer was used to detect the plasma TC level of chickens at 4 dpi (11 days old). Plasma TC **(E)**, HDL-C **(F)** and LDL-C **(G)** levels of chickens were detected with the automatic biochemical analyzer at 7 dpi (14 days of age). Transcript levels of DHCR24 **(H)** and CH25H **(I)** in chick kidney tissues were detected by qPCR at 7 dpi as previously described (Liu et al., 2018). Transcript levels of CH25H **(J)** and DHCR24 **(K)** in DF-1 cells were detected at the indicated time points after infection with 1 MOI of IBV. The cells were pretreated with Dex for 24 h before infection, and the Dex-untreated group was used as a control. L (DF-1), M (Vero) and N (H1299) were pretreated with 25HC at the indicated concentrations for 12 h, and the cells were then infected with IBV-Beaudette at an MOI of 1. Cells were collected at 12 hpi and subjected to western blot analysis. Cytotoxicity assays of 25HC in cells were based on previous studies (Zhang et al., 2019; Xie et al., 2019). **(O)** DF-1 cells were pretreated with cholesterol (10 μM) for 12 h, and cells were then infected with the IBV-Beaudette strain at an MOI of 1. Cells were collected at 12 hpi and subjected to western blot analysis; cholesterol-untreated cells were used as a control. **(P)**. The signal of protein bands was determined in Image J software.

To evaluate the correlation between IBV replication and stress induced by Dex, we performed RNA-seq and untargeted metabolomics to obtain gene expression profiles from DF-1 cells and metabolic profiles from the chicken kidney, respectively. The RNA-seq data showed that Dex-driven changes in DF-1 cells were significantly enriched in cholesterol biosynthetic pathways. As shown in **Figure 6A**, Dex treatment drove the overexpression of numerous genes associated with steroid biosynthesis, terpenoid backbone biosynthesis and fatty acid metabolism. The mRNA level of DHCR24, a key rate-limiting enzyme for cholesterol biosynthesis, was significantly up-regulated in DF-1 cells after Dex treatment for 6 h or 24 h (**Figures 6B, C**). To verify whether Dex increased cholesterol levels in chickens, we used an automatic biochemical analyzer to measure plasma TC, HDL-C and LDL-C levels. Dex significantly up-regulated plasma TC, HDL-C and LDL-C levels in chickens (**Figures 6D–G**). The data also indicated that the chickens infected with IBV at 7 dpi, compared with those in the mock group, showed significantly lower plasma TC and HDL-C levels (**Figures 6E, F**). In addition, the transcript levels of DHCR24 and CH25H, two key rate-limiting enzymes in cholesterol metabolism, were significantly down-regulated in chickens at 7 dpi (**Figures 6H, I**). Simultaneously, when DF-1 cells were invaded by IBV, the mRNA levels of CH25H and CHCR24 also showed significant differences according to Dex pretreatment (**Figures 6J, K**). As shown in **Figures 6L–N**, pretreatment with 25HC for 12 h significantly inhibited IBV replication, particularly in DF-1 cells. Moreover, the addition of exogenous cholesterol in DF-1 cells promoted the replication of IBV (**Figures 6O, P**). Thus, Dex treatment and IBV infection affected host cholesterol metabolism, and intrinsic link may exist between host cholesterol biosynthesis pathways and IBV infection.

## Discussion

Metabolic changes are the end-result of adaptive and defensive biochemical reactions that occur during infection (Kuang et al., 2020). In recent years, metabolomics has been widely used in chicken research to understand various host responses during viral infection (Liu et al., 2019; Xu et al., 2019). Examination of the metabolomes of the kidneys (Xu et al., 2019), bursa of fabricius (Kuang et al., 2020), lungs (Liu et al., 2019), plasma (Chen et al., 2021) and chicken cell lines (DF-1 and LMH) (Lin et al., 2020; Xu et al., 2022) has provided a new method for evaluating the regulation of metabolic pathways in virus-host interactions involving viruses such as IBV, NDV, ALV, IBDV and ILTV (Liu et al., 2019; Xu et al., 2019; Kuang et al., 2020; Lin et al., 2020; Chen et al., 2021; Xu et al., 2022). IBV is a pathogenic chicken coronavirus, which is a highly infectious in domestic chickens of all ages and types, and affects the respiratory, renal and reproductive systems (Amarasinghe et al., 2017; de Wit and Cook, 2019). Although considerable attention has been paid to coronaviruses, the metabolic regulatory mechanisms underlying chicken responses to IBV infection under stress conditions remain unclear. In the present study, we established a poultry stress model in 7-day-old chickens injected with Dex to elucidate the effects of stress on metabolic changes and IBV replication in chicken kidneys. We used an LC-MS/MS-based metabolomics method to examine differentially enriched metabolites in the kidneys and assessed the effects of Dex on chicken body weight, T cell subsets, serum cholesterol levels, cytokines and viral load. This work provides new insights into the interaction between the IBV and host metabolism under stress.

This study further revealed the changes in metabolites and connected pathways in chickens after IBV infection or treatment by Dex. Particular attention was paid to whether host metabolism manipulated by Dex affected the infective response to IBV in chickens. The results of PCA, OPLS-DA and hierarchical clustering revealed significant differences in the global metabolite profiles of controls and chickens infected with the IBV-QX strain or treated with Dex (**Figures 3A, B** and **Supplementary Figure S5**). Compared with the control, Dex-treatment showed a significantly differences in amino acids, steroid biosynthesis, fatty acid metabolism, butanoate metabolism, thiamine metabolism, histidine metabolism, arachidonic acid metabolism and linoleic acid metabolism (**Figures 4A, B**). In summary, the observed changes in these metabolites increase understanding of the intracellular reactions through which Dex affects IBV infection by manipulating host metabolic pathways. Previous studies have reported that GCs are important regulators of lipid metabolism, and chronic exposure to GCs promotes lipogenesis (John et al., 2016; Hu et al., 2018), In addition, cholesterol is a critical GC regulatory serum component (Yang et al., 2014). GCs impair HDL-mediated cholesterol efflux beyond increasing HDL cholesterol concentrations (Bouillet et al., 2020). This study supports previous observations in rats and chickens indicating that Dex treatment causes lipid accumulation (Cai et al., 2009; Wang et al., 2010; Cai et al., 2011; Wang et al., 2012), growth inhibition (Pan et al., 2019), changes in serum lipids (Malkawi et al., 2018) and hypercholesterolemia (Li et al., 2020), thus suggesting modulation of lipid metabolism disorders in chickens (Wang et al., 2010; Wang et al., 2012; Wang et al., 2012; Lv et al., 2018; Hu et al., 2020).

Our findings are broadly consistent with those of other comprehensive studies of human coronaviruses. A strong relationship between cholesterol and coronavirus replication has been widely documented in the literature (Thorp and Gallagher, 2004; Syed et al., 2010; Heaton and Randall, 2011; Blanc et al., 2013; Del Campo and Romero-Gómez, 2015; Guo et al., 2017; Meher et al., 2019; Lange et al., 2019; Cao et al., 2020; Radenkovic et al., 2020; Balgoma et al., 2020; Luquain-Costaz et al., 2020; Sanders et al., 2021; Cheng et al., 2021). Our previous study has also shown that cholesterol has an important role in coronavirus entry, membrane fusion and pathological syncytia formation; therefore cholesterol metabolic mechanisms may be promising drug targets for coronavirus infections (Dai et al., 2022). Herein, we obtained the gene expression profiles of DF-1 cells exposed to Dex for 24 h, and the RNA-seq data indicated that many genes associated with cholesterol biosynthesis were up-regulated by Dex. In view of this finding, we examined serum cholesterol levels in Dex-treated chickens and found that Dex treatment significantly upregulated serum cholesterol concentrations, including TC, HDL-C and LDL-C (**Figures 6D–G**). Previous research has shown that viral infections may cause host cells to alter the expression of cholesterol metabolizing enzymes and metabolites; similarly, cholesterol metabolism can also regulate host antiviral responses (Osuna-Ramos et al., 2018; Xiao et al., 2020). This notion was also supported by our data demonstrating that IBV infection significantly downregulated CH25H and DHCR24 transcript levels in chicks (**Figures 6H, I**). To support viral entry and increase virion production, IBV must consume host cholesterol. Similar results were also confirmed in chickens (**Figures 6E, F**). Moreover, the addition of exogenous cholesterol to DF-1 cells promoted IBV replication, but whether Dex promotion of IBV replication levels *in vivo* is associated with cholesterol biosynthesis remains to be further verified in subsequent experiments. In addition, most of the renal differentially enriched metabolites induced by Dex were involved in lipid metabolism pathways—notably cholesterol metabolism—which are very important for the replication of coronavirus IBV. The promotion of IBV replication in chicken kidney tissue may be associated with the up-regulation of cholesterol levels induced by Dex. The identification of these differentially enriched metabolites and metabolic pathways may lead to the development of drugs to combat coronavirus infections, thus further underscoring the importance of cholesterol in coronavirus infection. Therefore, weaponizing host cholesterol metabolism dysregulation against coronavirus infections may serve as an effective antiviral strategy (Sturley et al., 2020; Proto et al., 2021). Understanding the relationship between cholesterol biosynthesis and coronavirus infection will be an important direction for future research (Daniloski et al., 2021).

Dex has been investigated before in animal models and small clinical trials for infections with different coronaviruses, but the results have been mixed. Differences in timing and dose underlie many of the inconsistent and sometimes conflicting results across studies of GC therapy (Cain and Cidlowski, 2020). GCs are often used in combination with antiviral drugs to counteract painful inflammation and are known to inhibit the replication of some viruses (Lancz et al., 1990; Kim et al., 2017), However, this approach is controversial, because GC treatment has been suggested to increase the viral yield and susceptibility, thereby increasing lung lesions and increasing or prolonging shedding of viruses, such as SARS-CoV (Li et al., 2003), SIVs (Ali et al., 2013), HSV-1 (Erlandsson et al., 2002; Hara et al., 2009; Du et al., 2012; Pechan et al., 2014), MMTV (Parks et al., 1974; Indik et al., 2007), retroviruses (Solodushko et al., 2009), PRRSV-1 (Singleton et al., 2018) and FFV (Lee and Shin, 2018). Consequently, GC use is often hampered by the onset of adverse effects or resistance (Petta et al., 2016).

In summary, the metabolome profiles of chickens under stress induced by Dex and infected with the IBV-QX strain were analyzed to establish the characteristics by LC-MS/MS. The identification of these differentially enriched metabolites and metabolic pathways revealed that Dex treatment targets the cholesterol biosynthesis pathway in chicks and DF-1 cells. Host responses to IBV infection are also regulated by Dex. In addition, chicken growth and immune function are significantly inhibited by Dex. Although this study has some limitations, such as the use of a single tissue type and single time point for metabolite detection, it nonetheless provides a comprehensive analysis of host metabolic profile changes that occur during stress, and supplies new information for exploring the molecular regulatory mechanisms of avian stress. This article provides a comprehensive and in-depth understanding of poultry stress and should serve as a basis for further research to clarify the interaction between the virus and the host under stress.

## Data Availability Statement

The original contributions presented in the study are included in the article/**Supplementary Material**. Further inquiries can be directed to the corresponding authors.

## Ethics Statement

The animal study was reviewed and approved by Shanghai Veterinary Research Institute, Chinese Academy of Agricultural Sciences.

## Author Contributions

JD and XQ designed the experiments, analyzed the data, and wrote the manuscript. JD and HW performed the experiments. XQ, TL, CD, WL, LT, CS, and YS gave suggestions during the experiments. JD, XQ and CD revised the manuscript. All authors read and approved the final manuscript.

## Funding

This work was supported by the Shanghai Agriculture Applied Technology Development Program, China (G20180207), National Natural Science Foundation of China (grant No. 32030108), Shanghai Agriculture Applied Technology Development Program, China (2022-02-08-00-12-F01154) and Natural Science Foundation of Shanghai (grant Nos. 21ZR1476800 and 20ZR1469400).

## Conflict of Interest

The authors declare that the research was conducted in the absence of any commercial or financial relationships that could be construed as a potential conflict of interest.

## Publisher’s Note

All claims expressed in this article are solely those of the authors and do not necessarily represent those of their affiliated organizations, or those of the publisher, the editors and the reviewers. Any product that may be evaluated in this article, or claim that may be made by its manufacturer, is not guaranteed or endorsed by the publisher.

## Supplementary Material

Supplementary Table S1 Specific R package from this article.

Supplementary Table S2 Changes in body weight in chickens

Supplementary Table S3 The lists of differentially enriched metabolites in mock vs Dex (POS)

Supplementary Table S4 The lists of differentially enriched metabolites in mock vs Dex (NEG)

Supplementary Table S5 The lists of differentially enriched metabolites in mock vs IBV (POS)

Supplementary Table S6 The lists of differentially enriched metabolites in mock vs IBV (NEG)

Supplementary Table S7 The lists of differentially enriched metabolites in mock vs IBV-A-Dex (POS)

Supplementary Table S8 The lists of differentially enriched metabolites in mock vs IBV-A-Dex (NEG)

Supplementary Table S9 The lists of differentially enriched metabolites in mock vs IBV-P-Dex (POS)

Supplementary Table S10 The lists of differentially enriched metabolites in mock vs IBV-P-Dex (NEG)
