# Supplementary material for: Non-Targeted Metabolomic Analysis of Chicken Kidneys in Response to Coronavirus IBV Infection Under Stress Induced by Dexamethasone
Source: Front Cell Infect Microbiol. 2022 Jul 15;12:945865. doi: 10.3389/fcimb.2022.945865 (PMC9335950; doi:10.3389/fcimb.2022.945865)
Supplement: Supplementary file 3 [file DataSheet_3.docx]

**Fig.S1 Outline of experimental group design and detection analysis.**

**Fig.S2. Plasma TNF-α(C), IL-1β(D), IFN-γ(E), IFN-β(F) and IL-6(G) levels were assessed by ELISA kit（standard curve）.**


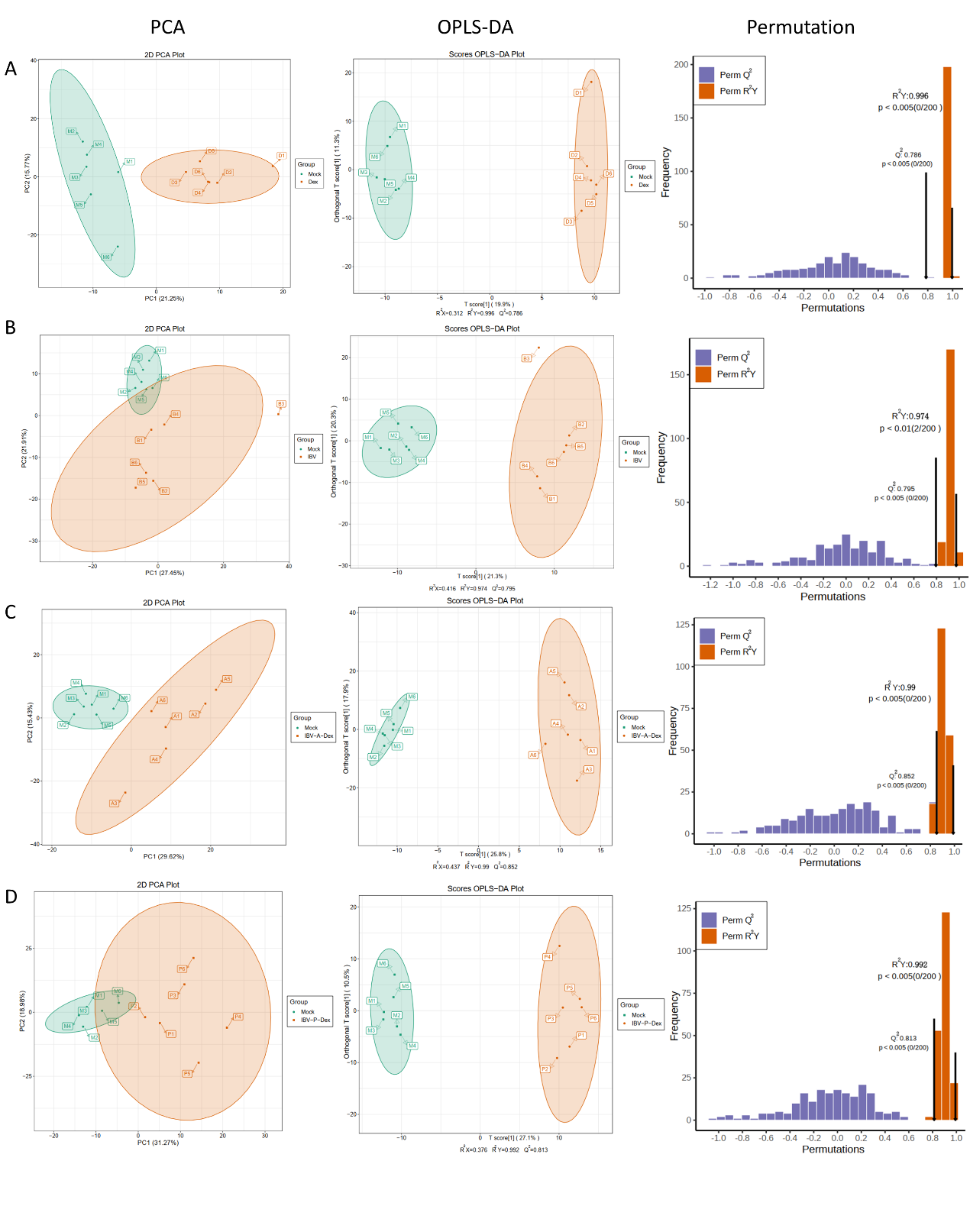


**Fig.S3 Multivariate Analysis of Chicken kidney Metabolites**

Metabolic profile of the mock and Dex group (A), IBV group (B), IBV-A-Dex group (C), and IBV-P-Dex group (D) visualized by principal component analysis (PCA), OPLS-DA analysis, and a permutation test. Ellipses represent 95% confidence intervals. (A), (B), (C), and (D) were derived from NEG.

R^2^


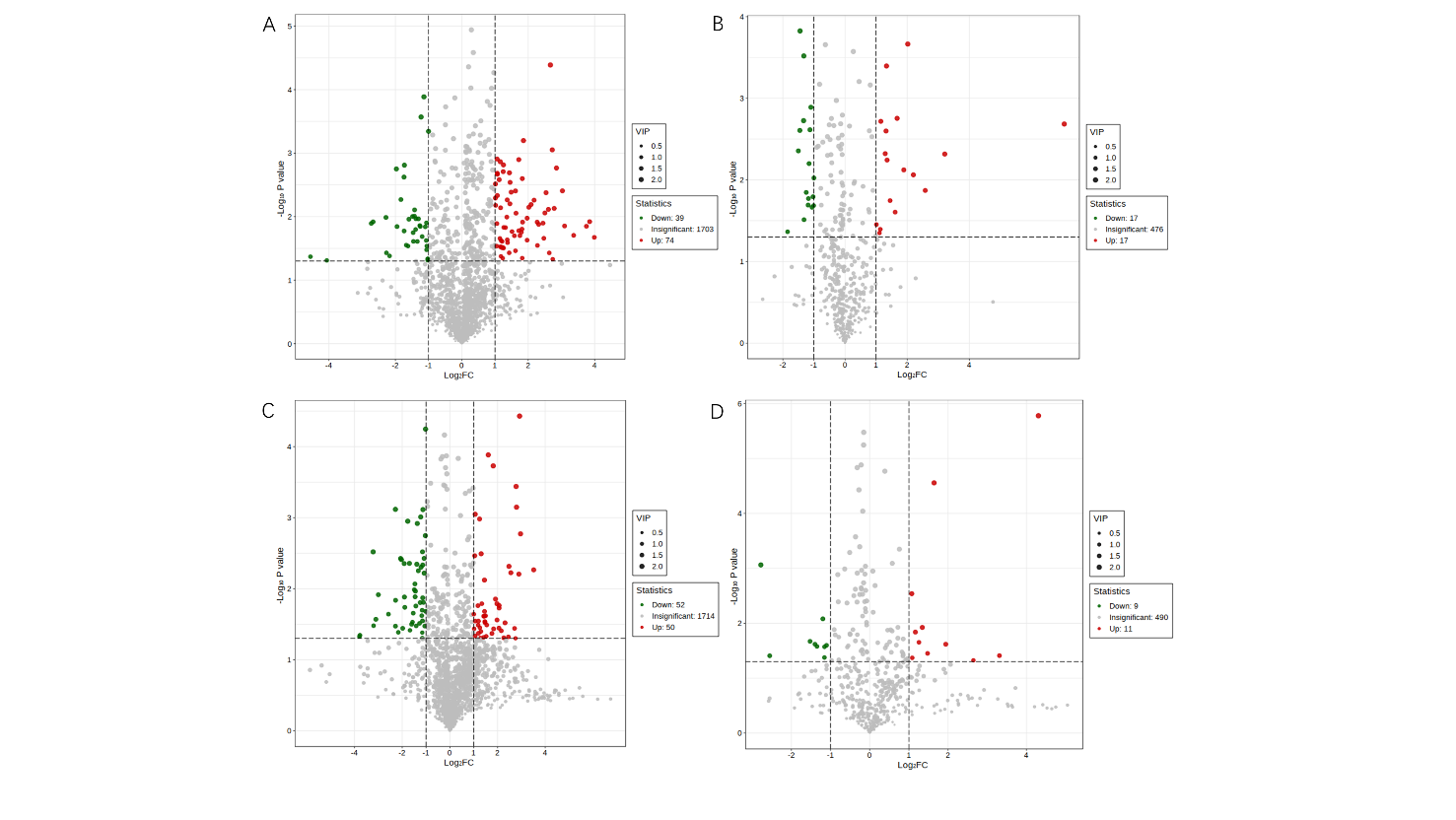


**Fig. S4 The volcano plots for the mock and Dex group or IBV group**

(A) and (B) are the volcano plots for the mock and Dex group. Each point in the volcanic map represents a metabolite. (A) was derived from POS and (B) from NEG. Red: Upregulation; blue: Downregulation; gray: Not significant.

(C) and (D) are the volcano plots for the mock and IBV group. Each point in the volcanic map represents a metabolite. (A) was derived from POS and (B) from NEG. Red: Upregulation; blue: Downregulation; gray: Not significant.

**Fig.S5** **Heatmap of hierarchical clustering analysis** **for the mock and Dex group or IBV group.**

Each column represents one sample, and each row represents one differential metabolite. The color of each cell represents the relative level of the differential metabolites. Red: Upregulation; green: Downregulation. (A) and (C) were derived from POS, (B) and (D)from NEG.

**Fig.S6 IBV-induced Metabolic responses of chick kidneys are affected by Dex.** Metabolites changes were further examined by constructing a Venn diagram (A, B). (A) was derived from POS and (B) from NEG. (C) and (D) are the volcano plots for the mock and IBV-P-Dex group. Each point in the volcanic map represents a metabolite. Red: Upregulation; blue: Downregulation; gray: Not significant. (C) was derived from POS and (D) from NEG. (E) and (F) are the KEGG pathway enrichment analysis based on differentially metabolites in IBV-P-Dex group relative to mock. (E) was derived from POS and (F) from NEG. Each bubble represents a metabolic pathway. The x-axis represents rich factor refers to the ratio of the number of differentially expressed metabolites in corresponding pathways to the total number of metabolites detected by this pathway. The y-axis represents the p-value of the metabolic pathway in the enrichment analysis, and the darker color of the bubble represents higher pathway enrichment.
